# Dietary Restriction and Exercise for Diabetic Patients with Chronic Kidney Disease: A Systematic Review

**DOI:** 10.1371/journal.pone.0113667

**Published:** 2014-11-25

**Authors:** Liesbeth Van Huffel, Charles R. V. Tomson, Johannes Ruige, Ionut Nistor, Wim Van Biesen, Davide Bolignano

**Affiliations:** 1 Department of Endocrinology, Ghent University Hospital, Ghent, Belgium; 2 European Renal Best Practice (ERBP), Ghent University Hospital, Ghent, Belgium; 3 The Richard Bright Kidney Unit, Southmead Hospital, Bristol, United Kingdom; 4 Nephrology Department, "Dr. C.I. Parhon" University Hospital, University of Medicine and Pharmacy "Gr. T. Popa," Iasi, Romania; 5 Renal Division, Ghent University Hospital, Ghent, Belgium; 6 CNR-Institute of Clinical Physiology, Reggio Calabria, Italy; University of Milan, Italy

## Abstract

***Background*:**

Obesity and sedentary lifestyle are major health problems and key features to develop cardiovascular disease. Data on the effects of lifestyle interventions in diabetics with chronic kidney disease (CKD) have been conflicting.

***Study Design*:**

Systematic review.

***Population*:**

Diabetes patients with CKD stage 3 to 5.

***Search Strategy and Sources*:**

Medline, Embase and Central were searched to identify papers.

***Intervention*:**

Effect of a negative energy balance on hard outcomes in diabetics with CKD.

***Outcomes*:**

Death, cardiovascular events, glycaemic control, kidney function, metabolic parameters and body composition.

***Results*:**

We retained 11 studies. There are insufficient data to evaluate the effect on mortality to promote negative energy balance. None of the studies reported a difference in incidence of Major Adverse Cardiovascular Events. Reduction of energy intake does not alter creatinine clearance but significantly reduces proteinuria (mean difference from −0.66 to −1.77 g/24 h). Interventions with combined exercise and diet resulted in a slower decline of eGFR (−9.2 vs. −20.7 mL/min over two year observation; p<0.001). Aerobic and resistance exercise reduced HbA1c (−0.51 (−0.87 to −0.14); p = 0.007 and −0.38 (−0.72 to −0.22); p = 0.038, respectively). Exercise interventions improve the overall functional status and quality of life in this subgroup. Aerobic exercise reduces BMI (−0.74% (−1.29 to −0.18); p = 0.009) and body weight (−2.2 kg (−3.9 to −0.6); p = 0.008). Resistance exercise reduces trunk fat mass (−0,7±0,1 vs. +0,8 kg ±0,1 kg; p = 0,001−0,005). In none of the studies did the intervention cause an increase in adverse events.

***Limitations*:**

All studies used a different intervention type and mixed patient groups.

***Conclusions*:**

There is insufficient evidence to evaluate the effect of negative energy balance interventions on mortality in diabetic patients with advanced CKD. Overall, these interventions have beneficial effects on glycaemic control, BMI and body composition, functional status and quality of life, and no harmful effects were observed.

## Introduction

Diabetes mellitus (DM) currently affects approximately 382 million people worldwide and its prevalence is expected to increase to 592 million by 2035 [1]. Diabetes is a prominent metabolic complication of obesity, which can be viewed as the result of a prolonged period of excess energy. Increasing energy expenditure by physical activity and reducing energy intake by caloric restriction are therefore mainstays of diabetes therapy to reduce cardiovascular risk and improve glycaemic control. The approach requires specific intensive programs and follow-up, which might have substantial impact on costs and resources. Diabetes is one of the leading causes of end stage kidney disease (ESKD) worldwide. Approximately 1 of 3 adults with diabetes has chronic kidney disease (CKD) and this proportion is steadily increasing in people with type 2 diabetes [2]. Management of diabetic patients with CKD is complex because of the multitude of systemic complications and often unstable clinical conditions, particularly in dialysis patients. Promoting energy expenditure or limiting energy intake in this population might therefore be challenging.

In diabetic patients with CKD, physical activity may have several potential benefits, including weight loss, improved muscle strength and cardiorespiratory fitness, reduction in blood pressure, and improved mood. Exercise during haemodialysis may also improve dialysis efficiency but can be associated with harms and increased risk of acute cardiovascular events. Similarly, provision of dietary advice to restrict caloric intake could have positive effects on several outcomes amongst patients with diabetes mellitus and CKD but could also lead to malnutrition, particularly in dialysis patients, and could decrease quality of life. In this systematic review we thus aimed to ascertain whether interventions focused at increasing energy expenditure or limiting energy intake may influence major outcomes, such as survival, cardiovascular events, kidney function, physical performance and quality of life in diabetics with CKD or on dialysis.

## Methods

### Data source and search strategy

MEDLINE, EMBASE and CENTRAL databases were searched for English-language articles without time restriction, through focused, high sensitive search strategies (Table S1). References from relevant studies and reviews published on the same topic were screened for supplementary articles.

### Study selection

We included all randomized or non-randomized trials, and single-arm, prospective or retrospective observational studies providing longitudinal data on the effect of energy expenditure on diabetic patients with clinically overt CKD, including ESKD on chronic renal replacement therapy. Studies were considered without restrictions on duration of follow-up. We planned to analyse studies dealing with diabetes (type 1 or 2) either as a cause of CKD or as a superimposed condition. Studies where a well-defined part of the population fulfilled the above criteria were included in the review. Interventions targeting energy control included lifestyle modifications, exercise, diet or multidisciplinary programs including two or more of these interventions. Outcomes of interest included all cause and cardiovascular mortality, major adverse cardiovascular events (MACE), glycaemic and blood pressure control, renal function (GFR, creatinine, proteinuria), body composition and weight, functional status, hospital admissions and quality of life. Studies were excluded if: 1) they did not include diabetic patients with CKD or CKD patients without diabetes; 2) they did not provide longitudinal data on the above mentioned outcomes after the planned intervention; 3) they examined (an) intervention(s) related to fluid (rather than energy) control. Case reports, reviews, editorials, letters and studies performed on children (age <18) or animals were excluded as well, although screened as potential sources of additional references. Relevant studies were selected by three authors (DB, CT and LVH). Data extraction was independently performed in duplicate by two authors (DB and LVH).

### Quality assessment

We used the Newcastle-Ottawa Scale to assess the study quality of observational studies. This scale considers a quality score calculated on the basis of three major items: Study participants (0 to 4 points), adjustment for confounding (0 to 2 points) or ascertainment of the exposure or outcome of interest (0 to 3 points) with a maximum score of 9 points which represents the highest methodological quality. The quality of RCTs was assessed using the checklist developed by the Cochrane Renal Group which evaluated the presence of potential selection bias (random sequence generation and allocation concealment), performance bias (blinding of investigators and participants), detection bias (blinding of outcome assessors), attrition bias (incomplete outcome data) and reporting bias (selective reporting).

### Data extraction and analysis

Data extraction and analysis were performed in duplicate by two reviewers independently (DB and LVH) and verified by a third one (CT). In studies considering mixed populations, the subgroup of patients with documented CKD and diabetes was selectively described only if corresponding data were available.

## Results

### Search results

**Figure 1:**
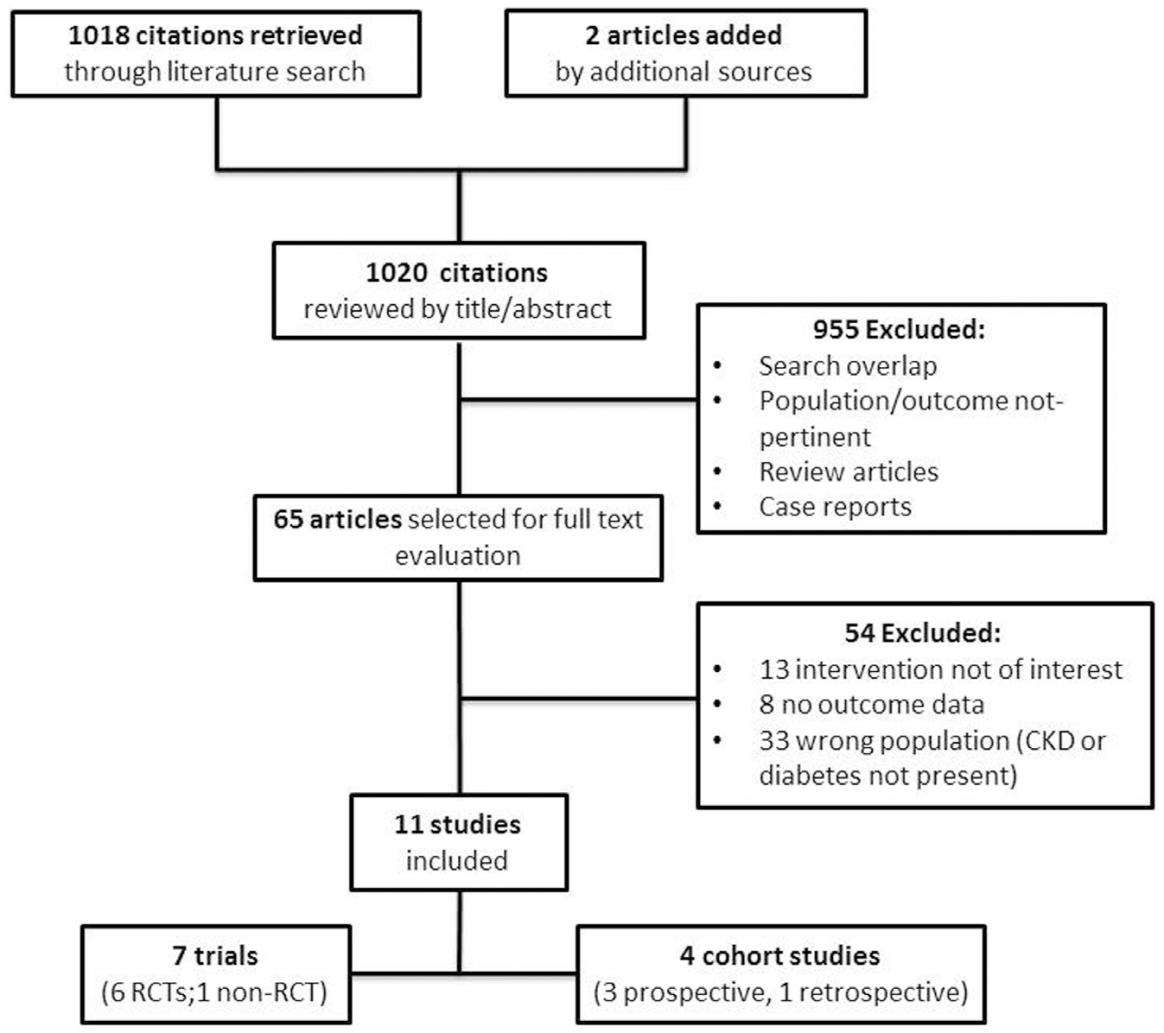
Flow of the study selection process.

The flow diagram of the selection process is depicted in **Figure 1**. One thousand-eighteen potentially relevant references were initially found. A total of nine hundred and fifty five citations were excluded because of search overlap, because they dealt with population without the inclusion criteria or because they did not contain original research. Case reports were also excluded as the information was regarded as too fragmentary. Two articles were added by additional sources. Amongst sixty-five studies selected for full text examination, fifty-four studies were excluded because: they dealt with interventions not affecting energy balance (n = 13); they did not provide longitudinal outcome data (n = 8); or because diabetes or CKD was not explicitly reported to be present in the study population (n = 33). A total of eleven studies was therefore reviewed in detail and included in the review. The main characteristics of these studies are summarized in **Table 1** **.**

**Table 1:** Summary of studies on energy control in diabetic subjects with CKD.

Authors	Year	Study type	Population	N =	Intervention	Outcome(s)	Results		Notes
Tawney et al.	2000	RCT	Diabetic hemodialysis patients	82	Individual counselling to exercise 30 min each day, for 6 months (household activities)		**Mean Score on KDQoL-SF (SD)**		Mixed group of diabetics and non-diabetics
						Physical functioning score	I: 47,3 (12,9) C: 49,9 (10,5)		
						QoL Mental component	I: 38,3 (10,5) C: 35,8 (8,8)		
						QoL Physical component	I: 62,3 (26,7) C: 48,5 (25,9) (p = 0,04 after adjusting matching variables and adequacy of dialysis)		
						Patient satisfaction	I: 61 (20,3) C: 67,4 (21,2)		
Castaneda et al.	2002	RCT	>55 years and type 2 diabetes of at least 3 years' duration	62	Progressive resistance training, 45 min 3 times/week for 16 weeks		**Mean (SE)**		Small groups and medication change during study
						SBP (mmHg)	I: 135,5 (3,3) C: 150,4 (3,9) P = 0,05		
						DBP (mmHg)	I: 69,2 (1,2) C: 70,8 (1,4) p = 0,52		
						HbA1c (%)	I: 7,6 (0,2) C: 8,3 (0,5) p = 0,01		
						FBG (mmol/L)	I: 7,9 (0,4) C: 8,9 (0,7) p = 0,34		
						Body weight (kg)	I: 79,5 (3,3) C: 79,4 (2,9) p = 0,89		
						Functional status (on physical activity score questionnaires)	I: 28,3 (0,9) C: 7,2 (2,8) p = 0,01		
Morales et al.	2003	RCT	Chronic proteinuric nephropathy of diabetic or non-diabetic cause, BMI>27 kg/m^2^, serum creatinine level less than 2 mg/dL	30	Energy reduction of 500 kcal/day, protein content adjusted to 1 to 1,2 g/kg/d, for 5 months		**Mean (SD):**		Small groups, combination of diabetics and non-diabetics.
						SBP (mmHg)	I: 138,5 (14,1) C: 140,4 (18,3)		
						DBP (mmHg)	I: 76,6 (8,8) C: 88,5 (11,1)		
						Serum creatinine (mg/dl)	I: 1,5 (0,8) C: 1,8 (0,6) p<0,05		
						Creatinine clearance (Cockroft-Gault formula)	I: 67 (34,1) C: 56 (19,9) p<0,05		
						Proteinuria (g/24 h)	I: 1,9 (1,4) C: 3,5 (2,1) p<0,05		
						Weight (kg)	I: 83,9 (10,9) C: 98 (16,4) p<0,05		
						BMI (kg/m^2^)	I: 31,6 (3,2) C: 35 (5,8) p<0,05		
Sigal et al.	2007	RCT	Type 2 diabetes - baseline HbA1c between 6,6% and 9,9%	251	15 to 20 min per session at 60% of HFmax to 45 min per session at 75% of the HFmax 3 times/week for 22 weeks		**Difference in change from baseline to 6 months (95% CI)**		Hospitalizations were elective and not related to intervention; hypoglycemia's were not severe.
						SBP (mmHg)	1(−3,6 to 5,7) p = 0,66		
						DBP (mmHg)	−1,5(−4,7 to 1,7) p = 0,36		
						HbA1c (%)	−0,51 (−0,87 to −0,14) p = 0,007		
						Body weight (kg)	−2,2(−3,9 to −0,6) p = 0,008		
						BMI (kg/m^2^)	−0,74(−1,29 to −0,18) p = 0,009		
						Hospital admissions intervention group (%)	3		
						Hypoglycemia intervention group (%)	7		
					7 different exercises on weight machines each session, progressive resistance training, 3 times/week for 22 weeks	SBP (mmHg)	−0,9(−5,4 to 3,7) p = 0,71		
						DBP (mmHg)	−1,4(−4,6 to 1,7) p = 0,37		
						HbA1c (%)	−0,38(−0,72 to −0,22) p = 0,038		
						Body weight (kg)	−0,7(−2,4 to 0,9) p = 0,36		
						BMI (kg/m^2^)	−0,26(−0,80 to 0,28) p = 0,35		
						Hospital admissions intervention group (%)	0		
						Hypoglycemia intervention group (%)	6		
					Combination of aerobic and resistance exercise intervention		**Compared with AE**	**Compared with RE**	
							**Mean difference (95% CI)**		
						SBP (mmHg)	1,3 (−3,4 to 1,7) p = 0,59	3,2 (−1,4 to 7,8) p = 0,168	
						DBP (mmHg)	1,7 (−1,5 to 5,0) p = 0,30	1,7 (−1,5 to 4,9) p = 0,30	
						HbA1c (%)	−0,46 (−0,83 to −0,09) p = 0,014	−0,59 (−0,95 to −0,23) p = 0,001	
						Body weight (kg)	0,0 (−1,6 to 1,7) p = 0,98	−1,5 (−3,1 to 0,1) p = 0,075	
						BMI (kg/m^2^)	0,03 (−0,58 to 0,53) p = 0,93	−0,50 (−1,05 to 0,04) p = 0,069	
						Hospital admissions intervention group (%)	**0**		
						Hypoglycemia intervention group (%)	**3**		
Leehey et al.	2009	RCT	Obese type 2 diabetes patients, CKD stage 2–4 with proteinuria	11	Aerobic walking exercise, increasing intensity, 30 á 40 min 3 times/week for 24 weeks		**Mean (SD)**		Small group of patients with significant baseline differences
						SBP (mmHg)	I: 113 (16) C: 136 (5)		
						DBP (mmHg)	I: 65 (10) C: 77 (8)		
						Creatinine clearance (mL/min)	I: 51 (26) C: 64 (10)		
						HbA1c (%)	I: 8,3 (2,4) C: 8,1 (3,7)		
						Mean duration exercise (min)	I: 10,2 (2,8) C: 6,6 (2,1)		
						Body Weight (kg)	I: 115 (23) C: 136 (20)		
						Proteinuria (mg/24 h)	I: 821 (1010) C: 490 (237)		
Chen et al.	2010	Quasi-randomized controlled trial	Stable CKD patients not on dialysis, selected by researcher	94	Exercise advice: 30 min per session, 3 to 5 times/week for 3 months, group sessions and individual guidance over telephone		**Mean (SD)**		Pre-test blood glucose values were used as the covariate
						Mean blood glucose (mg/dL)	I: 114,81 (30,28) C: 110,31 (25,58)		
MacLaughlin et al.	2010	Nonrandomized controlled trial	CKD patients with BMI>30 or BMI>28 kg/m^2^ with comorbidities (diabetes, hypertension, dyslipidemia), all eligible for kidney transplant	64	Individual diet and exercise plan, at least 3 times/week, with increasing time and intensity, Orlistat 3 times 120 mg/d, 24 months		**Mean (SD)**		Small groups
						SBP (mmHg)	I: 139 C: 139 (SD not reported)		
						DBP (mmHg)	I: 79 C: 84 (SD not reported)		
						Decrease in eGFR (MDRD formula) from baseline (mL/min) (only CKD 3–4)	I: −9,2 C: −20,7 (SD not reported) p<0,001		
						Body weight (kg)	I: 96 C: 101 (SD not reported) p<0,001		
						Accepted on kidney transplant list (%)	I: 35 C: 6		
						Number of transplants	I: 3 C: 1		
Matsuoka et al.	1991	Retrospective cohort study	Diabetes mellitus patients with diabetic nephropathy	13	Maintained daily physical activity		**Mean (SD)**		Small group, intervention not quantified
						SBP (mmHg)	I: 158 (27) C: 160 (11)		
						DBP (mmHg)	I: 86 (9) C: 85 (7)		
						Onset of nephrotic stage to dialysis (months)	I: 27,7 (13,9) C: 27,4 (14,7)		
						Maximum proteinuria to dialysis (months)	I: 6,0 (3,8) C: 8,6 (4,4)		
						Karnofsky Score	I: 82,7 (4,6) C: 77,1 (4,9) p<0,05		
Cappy et al.	1999	Before-after study	Hemodialysis patients with stable general and cardiovascular conditions	4	Training program consisting in a progressive, self-paced aerobic exercise. 20 to 40 min, 3 times/week for 12 months		**Mean % of change**		Small group
						SBP predialysis	−4%		
						DBP predialysis	−1%		
						Serum creatinine	0%		
						Serum glucose level	−16%		
Solerte et al.	1989	Prospective cohort study	Obese type 1 or 2 diabetic patients (BMI 33±1.6 kg/m^2^) with CKD (eGFR 66±13 mL/min)	24	52 weeks of hypocaloric diet (1410 kcal/day)		**Difference in change from baseline**		Small group No details about protein content of diet. Creatinine clearance change probably explained by less protein intake and muscle loss
						MAP (mmHg)	−9.7 (p<0.05)		
						Creatinine clearance (mL/min)	12 (p = 0.01)		
						Proteinuria (g/24 h)	−0.66 (p = 0.01)		
						BMI (kg/m^2^)	−7.3 (p<0.001)		
Saiti et al.	2005	Prospective cohort study	Overweight type 1 or 2 diabetic patients (BMI 30.4±5.3 kg/m^2^) with diabetic nephropathy (eGFR 40.6±17.9 mL/min; proteinuria 3.27±2.63 g/24 h)	22	4 weeks of 740–970 kcal per day diet		**Difference in change from baseline**		Short intervention, very restricted diet.Changes in creatinine and proteinuria were significantly related to those on BMI (r = 0.62 and 0.49 respectively).
						MAP (mmHg)	−7.4 (p<0.05)		
						Creatinine clearance (mL/min)	5.0 (NS)		
						Proteinuria (g/24 h)	−1.77 (p<0.0001)		
						HbA1c (%)	−0.43 (p<0.05)		
						BMI (kg/m^2^)	−2.2 (p<0.0001)		

**Legend:** **AE:** aerobic exercise; **BMI:** Body Mass Index; **CI:** Confidence Interval; **C:** Control; **CKD:** Chronic Kidney Disease; **DBP:** Diastolic Blood Pressure; **eGFR:** Estimated Glomerular Filtration Rate; **FBP:** Fasting Blood Glucose; **HbA1c:** Hemoglobin A1c; **Hfmax:** Maximum Heart Frequency; **I:** Intervention; **KDQoL-SF:** Kidney Disease Quality of Life Short Form; **MAP:** mean arterial pressure; **Min:** Minutes; **NS:** not significant; **QoL:** Quality of Life; **RCT:** Randomized Controlled Trial; **RE:** Resistance exercise; **SBP:** Systolic Blood Pressure; **SD:** Standard Deviation; **SE:** Standard Error.

### Study characteristics

#### Types of studies, populations and interventions

Six studies were randomized controlled trials [3], [4], [5], [6], [7], [8], one was a non-randomized controlled trial [9], three were prospective uncontrolled studies [10], [11], [12] and one was a retrospective study [13]. The number of participants included in each study ranged from 4 [10] to 251 [6].

The severity of renal function impairment was variable. MacLaughlin [9] included patients with CKD stage 3 to 5. Chen [4] included only CKD patients with eGFR>15 mL/min/1.73 m^2^. Leehey [5] included patients with eGFR between 15 and 90 mL/min/1,73 m^2^ and persistent proteinuria. In Morales [8] all patients had proteinuria and a serum creatinine level below 2 mg/dL. In Matsuoka [13] and Castaneda [3] all participants had CKD but the severity was not specified. The study of Sigal [6] excluded participants with a serum creatinine above 2.26 mg/dL but gave no additional information on kidney function. Two studies [7], [10] focused on hemodialysis patients. Solerte [11] included patients with an eGFR between 66 and 13 mL/min; Saiki [12] included participants with an eGFR between 40 and 17 mL/min, with proteinuria. The prevalence, type and duration of diabetes, as well as glycaemic control, differed between studies. Leehey [5] included obese participants with type 2 diabetes and a BMI above 30 kg/m^2^. Castaneda [3] selected type-2 diabetics with a disease history of at least 3 years and a mean HbA1c of 8.6%. Patients studied by Sigal [6] had type 2 diabetes of at least 6 months of duration, mean HbA1c was between 6.6 and 9.9% but none of the participants were treated with insulin. All patients of Solerte [11] were obese type 1 or 2 diabetics, mean HbA1c was not specified. In Saiki [12] all patients had diabetes type 1 or 2 with a mean HbA1c of 7.11%. In the study of Matsuoka [13] all the participants had diabetes with no further specification of the type or severity. In Tawney [7] 44% of the study population had diabetes and 29% were treated with insulin. In the study of Morales [8] 46% had diabetes of any type, with no further information on treatment regimens or severity. Fifty percent of the study population of Cappy [10] consisted of diabetics and 63% was insulin-dependent. The mean HbA1c was 6.88%±1.2%. Thirty-two and forty-one percent of the participants in the study of Maclaughlin [9] and Chen [4], respectively, were diabetics of either type. All studies excluded participants with any unstable clinical condition such as heart disease, cancer or rapidly progressive kidney disease. Five studies [3], [5], [6], [10], [13] involved physical exercise to improve energy balance. Three studies examined the effects of a dietary intervention [8], [11], [12], which consisted respectively of an energy reduction of 500 kcal per day and protein content adjusted to 1 to 1.2 g/kg/day [8], reduction of energy intake to 1410 kcal/day [11] and reduction to 11–19 kcal/kg/day [12]. In one study the participants got a combined dietary and aerobic exercise programme, in combination with behavioural therapy and a pharmacological intervention [9]. In two other studies [4], [7] the intervention consisted of exercise advice and counselling.

### Study quality

The study quality of RCTs was variable. In Sigal [6], central randomization was used, with allocation concealment before randomization and block sizes varied randomly between 4 and 8. In Tawney [7], patients were randomized to the intervention or control group with a frequency matching strategy based on age group (18 to 44, 45 to 64, and 65 years or older), sex, diabetes as cause of ESRD, and ethnicity. Data on random sequence generation and allocation concealment were not provided in any of the other RCTs included. Attrition bias was low in all studies with the exception of Tawney [7] where the overall drop-out rate was 17% although results were reported on a *per protocol* basis. In Sigal [6], to permit blinding of the research coordinator, the personal trainer rather than the research coordinator handled the randomization visit. Performance and detection bias were high in the remaining RCTs which were all not blinded. Reporting bias was low in all studies as all the outcomes defined were reported. The general quality of observational studies was low to moderate.

### Outcomes

#### Mortality

None of the studies reviewed included patient survival as a study outcome. In the study of Tawney [7], three deaths occurred in the group receiving the physical rehabilitation program vs. one in the control group but no details were available on whether these deaths were related to the intervention.

#### MACEs

None of the studies reviewed were specifically designed to evaluate MACEs as a study outcome. In the paper of Castaneda [3], three patients in the resistance exercise group had a minor episode of angina pectoris, of which one was hospitalised. One subject in the aerobic exercise group in the study of Sigal [6] was diagnosed with angina pectoris without need for hospitalisation.

#### Kidney function

Morales [8] reported no changes in mean serum creatinine or creatinine clearance after a dietary intervention with energy intake reduction of 500 kcal per day and limited protein intake. In a sub-population of MacLaughlin [9], a significantly lower decline in the eGFR was observed in the intervention- (weight management program by diet, aerobic exercise and behaviour therapy) than in the usual care-group (−9.2 vs. −20.7 mL/min; p<0.001). Saiki [12] showed no change in creatinine clearance after the dietary intervention, Solerte [11] showed a significant increase in eGFR after one year of dietary regimen (Δ15 mL/min; p = 0.01). This can be explained by loss of muscle mass or reduced intake of proteins since the value is an estimated GFR based on serum creatinine. Furthermore, 9 of 26 (35%) from the weight-management program group vs. 1 of 18 (6%) from the usual-care group who were otherwise eligible were accepted for kidney transplant listing. There were 3 transplants in the weight management program group (2 live related donor kidney transplants, 1 cadaveric donor kidney transplant) and 1 transplant in the usual-care group (live related donor kidney transplant). Conversely, no significant changes in eGFR were reported by Leehey [5] after an aerobic exercise intervention. In the retrospective cohort study of Matsuoka [13], maintenance of a physical active daily life did not affect progression to dialysis. Aerobic exercise regimens during and between hemodialysis sessions had no effect on serum creatinine levels or dialysis dose required [10]. In Morales [8], 24-hour proteinuria was significantly reduced at the end of the study in subjects undergoing a diet program as compared with the control group (1.9 g/24 h vs. 3.5 g/24 h; p<0.05). The dietary intervention in Solerte [11] and Saiki [12] also signficantly reduced proteinuria after the intervention compared with baseline (respectively −0.66 g/24 h; p = 0.01 and −1.77 g/24 h; p<0.0001). Conversely, no significant changes in this parameter were noticed after exercise or counselling interventions [5], [13].

#### Glycaemic control

As compared with no exercise, resistance exercise significantly reduced mean HbA1c (7.6±0.2% vs. 8.3±0.5%; p = 0.01) and mean fasting blood glucose levels [3]. One large RCT [6] demonstrated a significant reduction in HbA1c with aerobic and resistance exercise training (−0.51 (−0.87 to −0.14); p = 0.007 and −0.38 (−0.72 to −0.22); p = 0.038, respectively). Of note, this reduction was more pronounced when the aerobic and resistance training were combined (−0.46 (−0.83 to −0.09); p = 0.014 compared with aerobic exercise and −0.59 (−0.95 to −0.23); p = 0.01 compared with resistance exercise alone). Two small studies showed that low intensity aerobic exercise did not influence the mean blood glucose [10] or mean HbA1c [5]. Another larger study found that exercise advice alone without supervision or control of the exercise did not alter mean blood glucose levels [4].

A dietary intervention with very restricted calory intake of 4 weeks [12] significantly reduced mean HbA1c compared to baseline value (6.68±1.21 vs. 7.11±1.42; p<0.05).

#### Functional status

In Castaneda [3], the overall functional status as measured by a questionnaire, significantly improved after a resistance exercise intervention (p = 0.001). In Leehey [5], the mean exercise duration was more increased in the group undergoing aerobic exercise with respect to the control group, although this difference did not attain statistical significance. Studies exploring the effects of exercise advice [7] reported a significant improvement of the functional status (evaluated by a physical functioning score questionnaire) only after adjusting for matching variables and adequacy of dialysis (p = 0.04).

#### Quality of Life

In dialysis patients, exercise advice alone did not affect depression symptoms measured by the score on KDQoL-SF questionnaire [7]. In a very small retrospective cohort study, aerobic exercise significantly improved the overall quality of life (p<0.05; Karnofsky score for fitness in daily physical activity) in chronic ambulatory peritoneal dialysis patients and in hemodialysis patients [13].

#### Changes in body composition/weight

A large RCT testing a resistance exercise program demonstrated a significant reduction in trunk fat mass (−0,7±0,1 vs. +0,8±0,1 kg; p = 0.01–0,005) but not in body weight or waist circumference [3]. A larger RCT [6] reported significant changes in body weight, body mass index (BMI), waist circumference and fat mass when aerobic exercise was compared to no exercise. The mean BMI in the aerobic exercise group reduced by 0.74% (−1.29 to −0.18; p = 0.009) while the mean body weight was decreased by 2.2 kg (−3.9 to −0.6; p = 0.008). These changes were no longer significant in the resistance exercise group, but there was a trend for lower BMI when resistance was combined with aerobic exercise when compared with resistance exercise alone. In a small trial including only 11 participants, aerobic exercise did not result in significant changes in the mean body weight [5]. Dietary intervention with reduction of daily caloric intake by 500 kcal and limited protein intake significantly reduced mean body weight and BMI with respect to controls [8]. Two other dietary interventions [11], [12] significantly decreased BMI compared to baseline value (respectively −7,3 kg/m^2^; p<0.001 and −2.2 kg/m^2^; p<0.0001). Similar observations were reported after a combined intervention of an anti-obesity drug and individual diet and exercise plan [9].

#### Blood pressure

In one study [3], systolic blood pressure was significantly reduced after resistance exercise with respect to the control group (135.5±3.3 vs. 150.4±3.9 mmHg; p = 0.05). Two dietary interventions [11], [12] significantly reduced mean arterial pressure after intervention (respectively −9.7 mmHg; p<0.05 and −7.4 mmHg; p<0.05). No significant changes in both systolic and diastolic blood pressure were reported by other studies [5], [6], [8], [9], [10], [13].

#### Hospital admissions and adverse events

In the study of Sigal [6] two hospitalisations not related to the aerobic exercise program were recorded in the intervention group. In the trial of Castaneda [3], there were more hypoglycaemia episodes in the control- than in the intervention-group (7 vs. 5). In Cappy [10], two patients dropped out because of arthritic problems. In all the other studies, adverse events or hypoglycaemia episodes were not mentioned.

## Discussion

Results from our systematic review indicate that, overall, the evidence on the effects of energy control in diabetics with CKD, either achieved by increased energy expenditure or by reduced energy intake, is sparse and conflicting, and is only present for secondary outcomes. On the other hand, these interventions seem to be relatively safe, and seem to improve general well-being.

In the general CKD population the utility of energy control is debated. Obesity is an independent risk factor for the development and progression of CKD. Weight loss, particularly if achieved by bariatric surgery, reduces albuminuria, proteinuria and normalizes GFR in obese patients with non-terminal CKD [14]. However, no solid information is available on the long-term effects on CKD progression.

In dialysis patients reduced energy intake and low BMI are generally associated with an increased risk of morbidity and mortality [15], [16], [17]. Conversely, high BMI exerts a protective effect on survival [18], [19]. This so-called “dialysis obesity paradox” was clearly outlined in a large prospective cohort study [20] where even morbid obesity was associated with improved survival and reduced cardiovascular death. Moreover, weight loss was associated with increased cardiovascular and all-cause death, whereas weight gain showed a trend toward improved survival. This obesity paradox seems to be a consistent finding in many large observational trials in hemodialysis and peritoneal dialysis patients [21], but also in other chronic diseases like heart failure [22] and coronary heart disease [23]. However, it should be stressed that all these studies are observational, and do not distinguish an intentional weight loss from that induced by underlying inflammation or disease. In contrast, fat loss (rather than BMI decrease) by physical exercise should be considered as a positive endpoint. First because BMI does not seem to be an ideal marker for visceral obesity [21]. Postorino et al. [24] have shown that increased waist circumference and a higher waist/hip ratio have a stronger correlation with all-cause and cardiovascular mortality than BMI. Second, several studies demonstrated that a higher muscle mass exerts a protective effect regarding mortality [25], [26], [27], [28], and that visceral fat mass is detrimental. In addition, a high level of fitness and aerobic capacity is independently associated with increased survival, and the obesity paradox is mainly present in patients with low cardio-respiratory fitness [29], [30]. Accordingly, a weight loss of mainly fat tissue and a gain of muscle mass, as is obtained with physical exercise, should be favourable. Nevertheless, the obesity paradox in ESKD patients has biological plausibility and stays a point of discussion. Many explanations have been proposed, including a more stable hemodynamic status in obese individuals, reverse causation, survival bias, loss of lean body mass, cytokine and neurohormonal alternations and, probably most important, the overwhelming negative effect of the malnutrition inflammation complex on traditional cardiovascular risks [20], [31]. Patients with chronic kidney disease have an unacceptably high risk for premature death, mainly because of cardiovascular disease. As summarized by Stenvinkel et al. [32] emerging evidence suggests that endothelial dysfunction, oxidative stress, vascular calcification and inflammation are strongly interrelated and together play a major role in the initiation and progression of vascular disease in CKD. The question remains if weight loss or especially protein-wasting increases these processes and in this way augment the cardiovascular risk. This is an interesting subject for future research.

The increasing prevalence of overweight and obesity worldwide is alarming and has led to an unprecedented epidemic of type 2 diabetes [33]. Since sedentary lifestyle and unhealthy diet are the main causes of obesity, many efforts have focused on controlling energy balance. Dietary interventions, educational strategies and exercise regimens have shown to reduce weight and improve glycaemic control in type 2 diabetics [34]. A systematic review of studies on the general diabetic population identified 14 RCTs on exercise interventions. The studies investigated aerobic exercise and progressive resistance training and showed improved blood glucose control even without weight loss, decreased visceral adipose tissue and decreased plasma triglycerides. No study reported adverse events, but also none studied the effect on hard outcomes such as mortality or Major Adverse Cardiovascular Events [35]. A recently published large RCT investigated the effect on cardiovascular outcomes of an intensive lifestyle intervention in overweight or obese type 2 diabetes patients. Although the intervention had a beneficial effect on weight, glycaemic control, waist circumference and physical fitness, the rate of cardiovascular events was not affected [36]. There is enough evidence that exercise significantly improves glycaemic control while exercise advice alone did not produce significant benefits. Therefore, when implementing exercise in daily practice, the physician should make sure the patient is compliant and is performing the prescribed exercise. Aerobic exercise as well as a dietary program, significantly reduces BMI. Interestingly, resistance exercise may reduce trunk fat mass without decreasing BMI, therefore suggesting an increase in lean body mass. Data on the effect of energy control on quality of life suggest a beneficial effect, an observation in agreement with a recent systematic review of patients with CKD of various nature (also including diabetes) showing a significant improvement in the health-related QoL after any exercise training program [37].

There is too little evidence to draw conclusions on the effect of energy control on renal function and CKD progression. In one trial, 24 h proteinuria was significantly reduced by a combination of diet and exercise regimen [8] but the study population consisted of a mixed group of diabetic and non-diabetic patients, which may hamper the reliability of the conclusions. Two other trials with a dietary intervention showed a significant reduction of proteinuria [11], [12]. In the article of Solerte et al. [11], the mean creatinine clearance significantly augmented after one year of intervention. The studies were prospective cohort studies and included a small number of patients. A large systematic review that investigated the effect of weight loss on proteinuria in CKD patients came to the same conclusion: weight loss is associated with decreased proteinuria and microalbuminuria [38]. There were no data provided however on the effect on the progression of CKD. In this review [38], the interventions consisted of exercise, diet, medication and bariatric surgery and the population studied had CKD of mixed stages and included both diabetics and non-diabetics. Only one study of resistance exercise intervention reported a significant reduction in systolic blood pressure [3]. In the systematic review of Heiwe [37] in CKD patients, any type of exercise intervention significantly reduced systolic and diastolic blood pressure, although absolute changes were small. Two dietary interventions showed a small but significant reduction in mean arterial pressure [11], [12]. These results are similar to the results of a large exercise intervention trial in a general type 2 diabetes population: systolic and diastolic blood pressure significantly reduced after the intervention, but the absolute changes were again small [39]. On the other hand, another systematic review focusing on a general diabetic population [35] showed no effect of any type of exercise on blood pressure control.

Finally, there is no evidence available on the effect of energy control on mortality, MACEs or hospital admissions. Few studies reported these outcomes, but none indicated association with the intervention, suggesting that promoting energy control might not be harmful. Similarly, the Look AHEAD trial [36] did not demonstrate a substantial impact of intensive lifestyle interventions on (acute) cardiovascular events in obese type 2 DM patients although, as mentioned, such interventions improved HbA1c and BMI as well as quality of life, physical functioning and mobility.

No increase of hypoglycemic events was reported. This is of particular interest, because hypoglycemia is an established risk factor associated with cardiovascular complications such as coronary artery diseases, congestive heart failure, stroke and death [40], [41], [42], [43], [44]. In the ACCORD trial where conventional treatment was compared with intensive treatment in type 2 diabetes, a retrospective analysis showed a hazard ratio of 2.87 for all-cause mortality in patients with severe vs. non-severe hypoglycemia [45]. A recently published retrospective cohort study confirmed the higher risk of stroke and mortality in patients with hypoglycemia in CKD subjects [46]. In all of the studies included in this review, exercise and dietary interventions did not increase the number of hypoglycemia episodes.

Our review has some strengths and limitations. Strengths include a systematic search of medical databases, data extraction and analysis and study quality assessment made by two independent reviewers according to current methodological standards. However, although comprehensive search strategies focused on a specific population (diabetic CKD patients) and intervention (any approach targeting energy expenditure or energy intake) were implemented, publication bias cannot be excluded. In order to maximize the number of included studies we decided to adopt broad criteria, considering any paper including at least a subpopulation of diabetic patients with acknowledged renal dysfunction. Yet, in most studies diabetic (or CKD) patients often represented only a minor subpopulation of the whole study cohort and subgroup analyses according to CKD stage were not performed. This notably hampers the generalizability of findings to the whole diabetic CKD population. There was a high heterogeneity in the number of subjects enrolled, severity of diabetes (glycaemic control) and renal impairment, presence of co-morbidities, follow-up, type and duration of interventions (mostly exercise-based) which prevented us to perform data pooling. Furthermore, the majority of the included studies enrolled few patients and were powered to observe differences in surrogate rather than patient-centred outcomes. Due to this heterogeneity of the studies and the limited data available, it should be methodologically incorrect to perform a true meta-analysis. In conclusion, there is lack of evidence that energy control in diabetic CKD patients can improve hard patient centred outcomes (e.g. mortality, MACE, hospitalizations). There is however enough evidence that promoting energy expenditure or reducing energy intake (particularly by lifestyle interventions) might be useful for improving glycaemic control, BMI, body composition, quality of life and physical functioning. This might translate into better long-term outcomes, but future studies focusing on hard outcomes are needed. It is likely that the ‘dose’ of interventions to improve energy balance may have been inadequate in many of the studies we reviewed, with relatively small increases in energy expenditure on exercise programmes, and relatively small decreases in calorie intake in patients given dietary advice: if it were possible to persuade patients with diabetes and CKD to take enough exercise, for instance, more weight loss, improved fitness, and better long-term outcomes would be expected. Since there is also no evidence that these programs may harm, it would be reasonable to recommend energy control in those patients who are likely to benefits the most, like obese diabetic CKD patients. When introducing such measures in diabetic ESKD patients, we should provide professional advice and guidance to prevent malnutrition in this frail population.

## Supporting Information

Table S1 **Focused search strategy in CENTRAL and MEDLINE-EMBASE databases.**
(DOCX)

Checklist S1 **PRISMA checklist.**
(DOC)
