# Supplementary material for: Dietary Restriction and Exercise for Diabetic Patients with Chronic Kidney Disease: A Systematic Review
Source: PLoS One. 2014 Nov 25;9(11):e113667. doi: 10.1371/journal.pone.0113667 (PMC4244158; doi:10.1371/journal.pone.0113667)
Supplement: Table S1 — Focused search strategy in CENTRAL and MEDLINE-EMBASE databases. (DOCX) [file pone.0113667.s001.docx]

**Supplementary Table S1.** Focused search strategy in CENTRAL and MEDLINE-EMBASE databases

| **CENTRAL** |
| --- |
| - **dialysis:ti,ab,kw** - **h*emofiltration:ti,ab,kw** - **h*emodiafiltration:ti,ab,kw** - **(end-stage renal or end-stage kidney or endstage renal or endstage kidney):ti,ab,kw** - **(ESRF or ESKF or ESRD or ESKD):ti,ab,kw** - **(chronic kidney or chronic renal):ti,ab,kw** - **(CKF or CKD or CRF or CRD):ti,ab,kw** - **(CAPD or CCPD or APD):ti,ab,kw** - **(predialysis or pre-dialysis):ti,ab,kw** - **MeSH descriptor Kidney Failure, Chronic, this term only** - **MeSH descriptor Renal Replacement Therapy explode all trees** - **MeSH descriptor Renal Insufficiency, Chronic explode all trees** - **MeSH descriptor Diabetes Mellitus, this term only** - **MeSH descriptor Diabetes Mellitus, Type 1 explode all trees** - **MeSH descriptor Diabetes Mellitus, Type 2 explode all trees** - **MeSH descriptor Diabetic Nephropathies explode all trees** - **diabet*:ti,ab,kw** - **(niddm or iddm):ab,ti,kw** - **MeSH descriptor Exertion explode all trees** - **exercise:ti,ab,kw** - **(Physical and (Education or Training))** - **(physical next (training or activity or fitness or** - **rehabilitation)):ti,ab,kw** - **(resistance next (training or program*)):ti,ab,kw** - **(strength* and (muscle* or program* or training)):ti,ab,kw** - **kinesiotherapy:ti,ab,kw** - **energy intake:ti,ab,kw** - **explode Diet Therapy** - **explode diet** - **explode Feeding Behavior** - **nutrition*:ti,ab,kw** - **(nutri$ or diet$ or food or eat$):ti,ab,kw** |
| **MEDLINE** |
| - **Kidney Diseases/** - **exp Renal Replacement Therapy/** - **Renal Insufficiency/** - **exp Renal Insufficiency, Chronic/** - **dialysis.tw.** - **(hemodialysis or haemodialysis).tw.** - **(hemofiltration or haemofiltration).tw.** - **(hemodiafiltration or haemodiafiltration).tw.** - **(end-stage renal or end-stage kidney or endstage renal or endstage kidney).tw.** - **(ESRF or ESKF or ESRD or ESKD).tw.** - **(chronic kidney or chronic renal).tw.** - **(CKF or CKD or CRF or CRD).tw.** - **(CAPD or CCPD or APD).tw.** - **(predialysis or pre-dialysis).tw.** - **exp diabetes mellitus/** - **exp Diabetes Mellitus, Type 1/** - **exp Diabetes Mellitus, Type 2/** - **Diabetic Nephropathies/** - **diabet$.tw.** - **(niddm or iddm).tw.** - **energy intake/** - **exp Diet Therapy/** - **exp Feeding Behavior/** - **exp Diet/** - **nutrition*.tw.** - **(nutri$ or diet$ or food or eat$).tw.** - **Physical Exertion/** - **exp Exercise Therapy/** - **exp Exercise Test/** - **exp Physical Fitness/** - **exercise.tw.** - **(resistance training or resistance program$).tw.** - **(physical fitness or physical rehabilitation).tw.** - **(strength$ and (muscle or program$ or training)).tw.** - **(Physical and (Education or Training)).tw.** - **randomized controlled trial.pt.** - **controlled clinical trial.pt.** - **randomized.ab.** - **placebo.ab.** - **clinical trials as topic/** - **randomly.ab.** - **trial.ti.** - **exp Cohort studies/** |
